# Caffeine in the management of patients with headache

**DOI:** 10.1186/s10194-017-0806-2

**Published:** 2017-10-24

**Authors:** Richard B. Lipton, Hans-Christoph Diener, Matthew S. Robbins, Sandy Yacoub Garas, Ketu Patel

**Affiliations:** 1 Montefiore Headache Center, Department of Neurology, Albert Einstein College of Medicine, Louis and Dora Rousso Building, 1165 Morris Park Avenue, Room 332, Bronx, NY 10461 USA; 2 Department of Neurology, University Duisburg-Essen, Essen, Germany; 3 GlaxoSmithKline Consumer Healthcare, Parsippany, NJ USA

**Keywords:** Tension-type headache, Migraine, Caffeine, Acetaminophen, Acetylsalicylic acid, Ibuprofen

## Abstract

Caffeinated headache medications, either alone or in combination with other treatments, are widely used by patients with headache. Clinicians should be familiar with their use as well as the chemistry, pharmacology, dietary and medical sources, clinical benefits, and potential safety issues of caffeine. In this review, we consider the role of caffeine in the over-the-counter treatment of headache. The MEDLINE and Cochrane databases were searched by combining “caffeine” with the terms “headache,” “migraine,” and “tension-type.” Studies that were not placebo-controlled or that involved medications available only with a prescription, as well as those not assessing patients with migraine and/or tension-type headache (TTH), were excluded. Compared with analgesic medication alone, combinations of caffeine with analgesic medications, including acetaminophen, acetylsalicylic acid, and ibuprofen, showed significantly improved efficacy in the treatment of patients with TTH or migraine, with favorable tolerability in the vast majority of patients. The most common adverse events were nervousness (6.5%), nausea (4.3%), abdominal pain/discomfort (4.1%), and dizziness (3.2%). This review provides evidence for the role of caffeine as an analgesic adjuvant in the acute treatment of primary headache with over-the-counter drugs, caffeine doses of 130 mg enhance the efficacy of analgesics in TTH and doses of ≥100 mg enhance benefits in migraine. Additional studies are needed to assess the relationship between caffeine dosing and clinical benefits in patients with TTH and migraine.

## Review

Approximately 11% of adults worldwide have migraine [1, 2], a chronic neurologic disorder characterized by episodic attacks of head pain accompanied by autonomic symptoms as well as sensitivity to light and sounds. Migraine differs from tension-type headache (TTH), a more common condition that is characterized by mild to moderate headache with few or no associated symptoms [3]. Globally, at least 40% of adults meet criteria for TTH [2]. Both conditions account for substantial rates of absenteeism and lost productivity at work, home, and school, imposing a significant burden on both individuals and society [2].

Most people with migraine or TTH treat their acute headache episodes with medications. Worldwide, more than half of the patients with migraine (57%) or TTH (>80%) choose over-the-counter (OTC) medications to manage their condition, rather than prescription treatments [4, 5]. For migraine, this treatment approach might be partially explained by the wide spectrum of symptom severity from person-to-person and attack-to-attack, ranging from mild pain with little disability to severe pain with complete disability [6]. TTH is typically not associated with debilitating pain or functional impairment [2, 3].

In comparison with to OTC medications, prescription drugs are more costly, and are more likely to have more contraindications or undesirable side effects [7–9]. OTC medications can be used without the need for medical consultation [2]. In addition, a number of OTC agents have established efficacy in well-controlled trials in TTH and migraine, including acetaminophen (APAP) [10] and nonsteroidal anti-inflammatory drugs (NSAIDs), such as acetylsalicylic acid (ASA) [11] and ibuprofen (IBU) [12, 13]. There are also fixed combinations that combine analgesics with caffeine. Two such combinations that have demonstrated efficacy for patients with TTH or migraine are APAP 500 mg, ASA 500 mg, and caffeine 130 mg per 2-tablet dose (AAC-130) and APAP 400 mg, ASA 500 mg, and caffeine 100 mg per 2-tablet dose (AAC-100). As a result, evidence-based guidelines for the acute treatment of migraine and TTH recognize that OTC drugs are safe and effective treatment options for many patients [8, 14]. These guidelines also recognize that analgesics combined with caffeine, such as AAC-130 and AAC-100, have important advantages compared with other widely used OTCs, such as APAP, ASA, and IBU alone.

Caffeinated agents have been in worldwide use by headache patients, either alone or in combination with other treatments, for decades. Clinicians should be familiar with the therapeutic benefits and limitations associated with the use of these agents in clinical practice. Here we review the use of caffeine in primary headache management. We begin by reviewing the sources and consumption of caffeine as well as its chemical properties and pharmacology, clinical use, and safety. Next, we review studies of caffeine in the acute treatment of patients with TTH and migraine.

### Caffeine background

#### Sources and consumption

Caffeine is the most widely consumed psychoactive agent in the world [Citation removed]. It occurs naturally in more than 60 plant species, including tea, kola nuts, coffee beans, mate leaves, guarana plants, and cocoa nuts [15]. In humans, the most common sources of caffeine are dietary, as shown in Table 1 [16], although various medications also contain caffeine. Much of the caffeine used commercially is a byproduct of the production of decaffeinated coffee [17, 18].

**Table 1:** Selected sources of dietary and medical caffeine [16]

	Serving size (oz./mL)		Caffeine (mg)
Dietary			
FDA limit for cola and pepper soft drinks	12	355	71 (200 ppm)
Black tea, brewed for 3 min	8	237	30–80
Green tea, brewed for 3 min	8	237	35–60
Medical			
Midol Complete	2 caplets		120
Bayer Back & Body	2 caplets		65
Anacin	2 tablets		64
Excedrin ES	2 tablets		130 mg
Excedrin Migraine	2 tablets		130 mg
Excedrin Tension Headache	2 tablets		130 mg

Worldwide, approximately 80% of adults consume a caffeinated product every day [19], although daily mean consumption rates vary considerably by country. China (16 mg) and Kenya (50 mg) are at the low end of caffeine consumption and Brazil (300 mg) and Denmark (390) are at the high end [15]. In the United States, the vast majority of adults are regular caffeine users (90%), and estimates of daily consumption range from 168 to 220 mg [15].

#### Chemistry and pharmacology

**Fig. 1:**
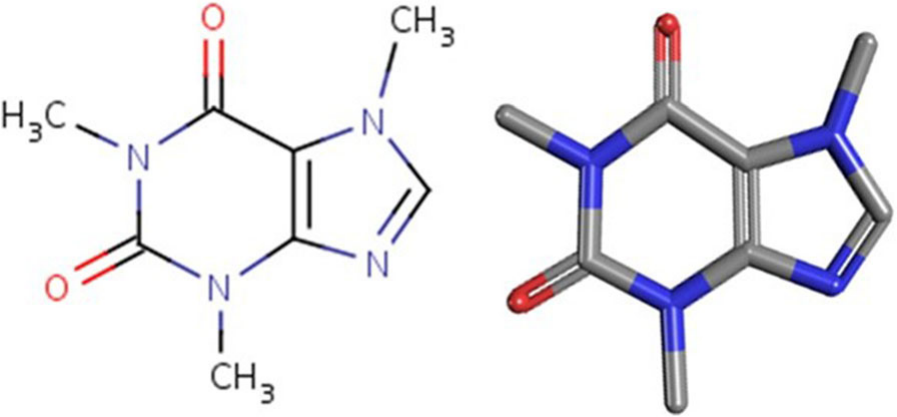
Molecular structure of caffeine [18]. The molecular structure of caffeine indicates that it is a part of a group of compounds called trimethyl xanthines, which also includes theobromine and theophylline

Caffeine, the common name for 1,3,7-trimethylxanthine, is a purine alkaloid with the molecular formula C_8_H_10_N_4_O_2_, a molecular weight of 194.1906 g/mol, and the molecular structure of a trimethyl xanthine (Fig. 1) [18]. Pure caffeine takes the form of white, prismatic crystals; it is odorless, has a slightly bitter taste, and is slightly acidic, with a pH of 6.9 (1% solution) [18].

Caffeine is completely absorbed by the intestinal tract (ie, its bioavailability is 100%) [20] and it is highly soluble in water as well as a range of non-polar organic solvents [21]. When administered orally, caffeine takes 30–120 min to reach maximum plasma concentration, although food can slow the absorption process [15]. Caffeine crosses both the blood-brain and placental barriers [22] and it is secreted in human milk, saliva, bile, and semen [21]. About 95% of a caffeine dose is metabolized in the liver via cytochrome P450 (CYP1A2 demethylation), which converts it into paraxanthine (85%), theobromine (10%) and theophylline (5%) [23, 24]. Less than 2% of a caffeine dose is eliminated unchanged in urine [23]. In adults, caffeine’s elimination half-life is 3–5 h, but it is doubled in smokers and non-coffee drinkers; in newborns, the half-life of caffeine is estimated to be 100 h [21, 23].

The effects of caffeine on nociception are primarily attributed to its nonselective antagonism of the adenosine A_1_, A_2A_, and A_2B_ receptors (as caffeine and adenosine possess a similar molecular structure) and its slightly lower-affinity antagonism for the adenosine A_3_ receptor [23, 25]. Through antagonism of the A_2A_, and A_2B_ receptors, adenosine induces vasodilation [26, 27]. To combat increased antagonism of these receptors and vasodilation in chronic caffeine users, adenosine receptors are upregulated resulting in vascoconstrictive effects [28]. Additionally, the mechanism by which the adjuvant effects of caffeine occur is not well established although studies have shown that caffeine does not reduce prostaglandin synthesis, a previously suggested mechanism of action [29].

Caffeine dosing influences its pharmacodynamic effects. Higher doses (75–100 mg kg^−1^) involve central noradrenergic mechanisms, medium to high doses (10–35 mg kg^−1^) activate central amine systems, and low doses (5 mg kg^−1^) interacting with central cholinergic mechanisms [30]. At therapeutic doses (ie, 100 times those typical in dietary consumption), caffeine can also induce phosphodiesterase inhibition, Ca^2+^ release, and GABA_A_ receptor blockade [23, 30].

#### Medical applications

Among patients with headache conditions, caffeine is used as an analgesic adjuvant. Analgesic adjuvants do not relieve pain by themselves, but augment the action of a known analgesic [31]. Factorial studies that examined the effect of analgesics alone, caffeine alone, and the combination of an analgesic and caffeine are required to assess its role as an analgesic adjuvant. In a 1984 meta-analysis of 30 such studies with more than 10,000 patients with postpartum uterine cramping, episiotomy pain, postsurgical pain, or headache, Laska et al., compared AAC-130 with APAP, ASA, and APAP + ASA to assess the therapeutic effect of caffeine as an analgesic adjuvant [32]. The estimated relative potency was 1.41 (95% CI; 1.23–1.63), indicating that caffeine significantly enhanced the analgesic effect of ASA, APAP, or a combination of the two by about 40% [32].

There is some evidence that caffeine monotherapy is associated with more pain relief than placebo in treating samples that include either migraine alone or persons with migraine or TTH [33, 34]. There is also limited evidence for the therapeutic use of caffeine monotherapy in patients with other primary and secondary headaches. For example, in patients with post-dural puncture headache (PDPH), studies have shown that acute use of 300 mg (oral) and 500 mg (intravenous) caffeine can provide relief [35]. A Cochrane review concluded that these doses of caffeine were superior to placebo at reducing the proportion of participants with PDPH persistence, as well as those needing supplementary interventions [35]. However, lower doses of caffeine (75 mg or 125 mg) combined with APAP 500 mg given every 6 h for 3 days have been shown to be ineffective for prophylaxis of PDPH [35], and a review of controlled and open-label studies investigating prophylaxis and treatment of PDPH with oral or IV caffeine cited methodologically weak trials and a paucity of reliable results in concluding that the use of caffeine as an antinociceptive agent for prevention or treatment of patients with PDPH is insufficiently supported by the available pharmacological and clinical evidence [36]. In patients with hypnic headache, an uncommon condition in elderly patients featuring stereotyped attacks of nocturnal headache for which outcomes from controlled studies are lacking, a review of data from observational studies found that the most effective acute treatment is caffeine (given either as 40–60 mg tablets or as a cup of coffee) [37].

When combined with analgesics, caffeine produces a range of clinical effects in these patients. Caffeine promotes the absorption of analgesics, including APAP, ASA, and IBU [38–40]. This effect is attributed to rapid lowering of gastric pH. At low doses (≤10 mg kg^−1^), caffeine has been shown to inhibit the antinociceptive effects of APAP [41], amitriptyline [42], carbamazepine and oxcarbazepine [43]. At higher doses (10–35 mg kg^−1^), caffeine enhances pain relief with APAP [44, 45] and several different NSAIDs [46, 47]. Meta-analyses of caffeine combined with APAP, ASA, or IBU have found only weak adjuvant effects in patients with postoperative pain [48, 49].

Coadministration of caffeine can have other effects that are relevant for patients taking analgesics, including interfering with the effectiveness of some therapies. For instance, a 200-mg caffeine dose can inhibit the analgesic effects of transcutaneous electrical nerve stimulation [50], and consuming caffeine within 2–4 h of intravenous adenosine therapy for paroxysmal supraventricular tachycardia has been shown to necessitate the use of higher adenosine doses [51].

Caffeine has a wide range of physiologic effects that are unrelated to the direct treatment of headache pain but may influence improvement in headache symptoms. Multiple studies have shown that caffeine can enhance mood, alertness [23, 52–55]; exercise performance [56, 57]; the speed at which information is processed, awareness, attention, and reaction time [58, 59].

Caffeine also increases gastric motility, as measured by pressure waves and propagated contractions in transverse/descending colon. The magnitude of the effect of caffeinated coffee is similar to eating a meal, 60% stronger than water, and 23% stronger than decaffeinated coffee [60]. Pharmacokinetic studies have shown that the absorption of caffeine is related to gastric emptying [61]. This effect of caffeine has important clinical implications for people with migraines, who may experience reductions in gastric motility both during and between attacks [62]. This reduction in gastric motility slows the absorption of acute medications and reduces their effectiveness [63]. In addition, widely used migraine-specific therapies, including the oral and injectable formulations of sumatriptan, significantly decrease gastric motility in healthy volunteers [64–68], potentially exacerbating disease-related gastroparesis and, perhaps, decreasing or delaying oral absorption of triptans resulting in lower efficacy in more advanced attacks of migraine.

#### Safety

In the United States, the Food and Drug Administration considers caffeine a substance generally recognized as safe (GRAS) when used in 1) cola-type beverages in accordance with good manufacturing practice and 2) stimulant drug products [69–71]. The caffeine contained in OTC analgesics is limited to a dose of 64–65 mg per tablet though two tablet doses are common [69–72]. In clinical trials, caffeinated analgesics were generally well tolerated and caused the types of mild and transient adverse events (eg, upset stomach) that might be expected from single doses of analgesics at OTC doses [33, 34, 73, 74]. For example, one meta-analysis estimated that adding caffeine to analgesics significantly increased the number of patients who experienced nervousness and dizziness (relative risk 1.60) [75].

Studies also suggest that caffeine stimulates gastric acid production and relaxes the lower esophageal sphincter [76], increasing the risk of gastric and duodenal ulcers [77, 78] as well as gastroesophageal reflux disease [79]. However, a meta-analysis of studies enrolling more than 8000 healthy subjects detected no significant association between coffee intake and acid-related upper gastroduodenal ulcer diseases, including gastric and duodenal ulcers, reflux esophagitis, and non-erosive reflux disease [80]. The authors speculated that the known protective effects of coffee (relaxing effect, antioxidant effect, phytochemical effects) might outweigh the risks of increased gastric acid secretion [80].

When used in patients with renal failure, caffeine requires no dose adjustment [12]. However, as a matter of good clinical judgment, it is prudent to consider all analgesics potentially nephrotoxic and to counsel against excessive, protracted use [46, 81]. Excessive use of phenacetin-containing analgesics can cause renal papillary necrosis and interstitial nephritis, but there is no convincing evidence that nonphenacetin-containing analgesics, including acetaminophen (APAP), aspirin (ASA), and mixtures of these two compounds, or NSAIDs, are associated with pathologically or clinically defined renal disease [82].

The role of caffeine in the treatment of migraine is complex. Caffeine combination products are useful in treating migraine and other headache types [33–35]. However, an association between caffeine intake and acute migraines has been observed [83]. Caffeine withdrawal may precipitate headache in the short term (NEJM) while cessation of caffeine may be beneficial. This suggests that complete abstinence of caffeine, like abrupt discontinuation of analgesics for those with MOH headaches [84], may be beneficial to those suffering from migraines [83]. Furthermore, overuse of APAP/caffeine/ASA has been shown to affect regional brain glucose metabolism in patients with chronic migraine [85].

#### Potential for overuse

Frequent use of analgesics is an important health problem [84], and medication overuse by patients with episodic headache conditions is associated with the development of chronic headache conditions [86–89]. Avoiding medication overuse is an important aspect of headache care [3]; once established, medication overuse may be difficult to treat, and recidivism is common [90]. Some research has identified dietary and medicinal caffeine consumption, specifically OTC caffeine combinations, as a modest risk factor for progression from episodic to chronic headache [91, 92]. After adjustment for demographic factors, primary headache type, and number of medications taken, the association of caffeine-containing combinations, and opioid-containing combinations as a risk factor for migraine progression was attenuated [92]. Additional support for the lack of positive association between risk of migraine chronicity and the appropriate use of caffeine-analgesic combinations came from the American Migraine Prevalence and Prevention study, which reported that AAC-130 had no association with development of chronic migraine. After adjusting for headache frequency and medication use, AAC-130 was not associated with an increased risk of chronic migraine onset (OR = 0.87, 95% CI 0.64,1.19) [84]. Acute treatment guidelines recommend that in all cases and with all acute medications, migraineurs should limit medication use to 2–3 headache days per week [8]—advice that may apply equally to patients with TTH [93].

Chronic intake of caffeine can cause a dependence syndrome [94, 95] that has also been positively associated with a history of cigarette smoking and problematic alcohol use [96]. However, compulsive drug-seeking behavior has never been reported among caffeine-dependent persons, and reports of deliberate caffeine abuse are uncommon [97]. It has been suggested that the prevalence of caffeine dependence may increase as a result of the promotion of caffeinated energy drinks to adolescents [98], and cases of intoxication—characterized by nausea/vomiting, tachycardia, hypertension, agitation, and dizziness—have been reported [99]. Caffeinated energy drinks have also been associated with seizures [100], stroke [101], and, rarely, death [102]. These drinks are also frequently combined with alcohol, a combination that has been found to increase alcohol consumption in laboratory animals [103]. Perhaps as a consequence, consumption of alcohol mixed with caffeine has been found to be associated with high-risk behaviors such as binge drinking [103].

#### Tolerance

Tolerance to the effects of caffeine on blood pressure, heart rate, diuresis, adrenaline and noradrenaline plasma levels, renin activity, and sleep patterns generally occurs within a few days [22], and regular daily doses of 750–1200 mg can lead to tolerance to its subjective, pressor, and neuroendocrine effects [104–106]. In addition, a withdrawal syndrome characterized by headache and sometimes other symptoms (ie, decreased cognitive performance, nausea/vomiting) may occur 12 to 24 h after stopping chronic consumption of caffeine in about 50% of patients [22, 31].

Multiple factors, including heritability [107] may contribute to an individual’s susceptibility to caffeine withdrawal [108]. The amount of caffeine consumption required to trigger withdrawal varies; while formal diagnostic criteria for caffeine withdrawal headache require daily consumption of ≥200 mg for at least 2 weeks [1], symptoms have been observed after sudden termination of caffeine exposures of 300 mg for 3 days and 100 mg for 7 days [109].

Some evidence suggests that caffeine withdrawal may play a role in triggering migraine attacks [110], especially weekend attacks [111]. Data also suggests caffeine withdrawal plays a role in dialysis headaches, a frequent complication of hemodialysis [112]. In addition, postoperative headaches may be partially due to caffeine withdrawal triggered by the abstinence typically required before surgical procedures [113]. Caffeine withdrawal headache can be minimized by a staged reduction and elimination of caffeine intake [97].

## Review of randomized trials of OTC analgesics combined with caffeine in the acute treatment of migraine and TTH

### Methods

To review literature relating to the use of caffeine by patients with TTH or migraine, research published between January 1966 and November 2016 was retrieved from the MEDLINE and Cochrane databases by combining “caffeine” with each of the following search terms: “headache” (597), “migraine” (365), and “tension-type” (52). Studies that were not placebo-controlled or that involved medications available only with a prescription (eg, butalbital- and ergotamine-caffeine combinations), as well as those not assessing patients with migraine and/or TTH, were excluded. A total of 7 placebo-controlled and factorial trials (2 in patients with TTH, 2 in patients with migraine, and 3 in mixed populations) were identified for analysis.

### Results

#### Efficacy in TTH

In 4 randomized, double-blind, 2-period crossover studies involving 1900 patients with TTH, Migliardi et al. compared AAC-130 with APAP alone and placebo [74]. Efficacy endpoints included pain intensity difference (PID), pain relief (PAR), and total pain relief (TOTPAR). AAC-130 provided significantly greater PID and PAR than APAP alone (*P* < .001), and both active treatments were superior to placebo (*P* < .001). As shown in Table 2, the magnitude of the adjuvant effect from caffeine for the summed pain intensity difference (SPID), %SPID, and TOTPAR was between 76% and 97%. For measures of peak analgesia and duration of analgesia, the effects were 63–85% greater than the net analgesic effect of APAP monotherapy [74]. Compared with APAP 1000 mg plus placebo, AAC provided significantly superior total pain relief at 4-h postdose (*P* < .001 vs. APAP and placebo) [74].

**Table 2:** Pooled results from 2 TTH trials in patients over 4 h for AAC-130, APAP, and placebo [74]

Measure	AAC-130 (*n* = 1369)	APAP (*n* = 1376)	Placebo (*n* = 689)
SPID	5.30 ± 0.06^a,b^	4.70 ± 0.07^b^	3.95 ± 0.10
%SPID	57.6 ± 0.7^a,b^	50.6 ± 0.7^b^	42.7 ± 1.0
Maximum PID	1.89 ± 0.02^a,b^	1.74 ± 0.02^b^	1.53 ± 0.03
TOTPAR	10.52 ± 0.10^a,b^	9.38 ± 0.10^b^	8.20 ± 0.16
Maximum PAR	3.42 ± 0.02^a,b^	3.14 ± 0.03^b^	2.81 ± 0.04
Time to pain at least half gone (hours)	3.01 ± 0.03^a,b^	2.73 ± 0.03^b^	2.38 ± 0.05

AAC-130, acetaminophen 500 mg, aspirin 500 mg, and caffeine 130 mg per 2-tablet dose; APAP, acetaminophen 1000 mg per 2-tablet dose; SPID, sum of pain intensity differences; PID, pain intensity difference from baseline; TOTAR, total pain relief; PAR, pain relief

Values are mean ± standard error

^a^ *P* < 0.05 vs APAP

^b^ *P* < 0.05 vs placebo

Because Migliardi et al. only restricted consumption of dietary caffeine (ie, coffee, tea, soda, chocolate) during the 4-h study period and found significant adjuvancy at the high and low ends of consumption, they concluded that the adjuvant effect was not influenced by patients’ usual caffeine consumption. Since the magnitude of caffeine’s adjuvant effect was larger for headache than in other forms of pain (ie, episiotomy, postsurgical, etc.) [32], caffeine-containing analgesic combinations may be particularly effective for headache pain [74].

In a randomized, double-blind, parallel-group, multicenter, single-dose, 4-arm, placebo- and active-controlled study, 301 patients with TTH treated a single attack with a 2-tablet dose of IBU 400 mg + caffeine 200 mg (IBC-100), IBU 400 mg alone, caffeine 200 mg alone, or placebo [34]. IBC-100 demonstrated significantly shorter times to meaningful improvement in headache relief than IBU alone or placebo; significantly greater total analgesia than IBU alone, caffeine alone, or placebo; and significantly greater peak relief than IBU alone, caffeine alone, or placebo (*P* < .05). Significantly more subjects obtained meaningful headache relief with IBC-100 than with IBU alone or placebo (*P* < .05). IBC-100 provided greater analgesic effectiveness than either component alone [51].

#### Efficacy in migraine

The efficacy of AAC-130 in migraine was established by Lipton et al. in 3 double-blind, placebo-controlled, parallel-group studies that randomized a total of 1220 migraineurs, excluding subjects who vomited more than 20% of the time or who usually required bed rest [73]. At 2-h postdose, pain-free rates were 21% for AAC-130 and 7% for placebo, and headache response (pain reduced from moderate or severe to mild or none) was 59% for AAC-130 and 33% for placebo. At 6-h postdose, 51% of patients treated with AAC-130 were pain-free compared with 24% of placebo-treated patients, and 79% of patients treated with AAC had a headache response versus 52% of those treated with placebo (*P* < .001 for all comparisons). The significant advantages of AAC-130 over placebo were also seen at 2- and 6-h postdose for relief of pain, associated symptoms, and disability (Table 3) [73]. Vomiting was less frequent in those treated with AAC-130 (0.2%) than placebo-treated patients (1.6%; *P* = .01) [73]. Although this study confirmed that a fixed combination containing caffeine could effectively treat a select population of migraineurs, the adjuvant effect of caffeine could not be assessed because a factorial design was not used.

**Table 3:** Pooled results from 3 migraine trials: AAC-130 versus placebo for migraine at 2 and 6 h postdose [73]

	AAC-130 *n* = 602	Placebo *n* = 618
Results at 2 hours^a^		
PID	1.0^b^	0.4
Proportion pain-free (%)	21^b^	7
Proportion nausea-free (%)	63^c^	56
Proportion photophobia-free (%)	35^b^	17
Proportion phonophobia-free (%)	37^b^	19
Proportion with little or no functional disability (%)	59^b^	34
Results at 6 h		
PID	1.4^b^	0.6
Proportion pain-free (%)	51^b^	23
Without nausea (%)	74^b^	60
Without photophobia (%)	58^b^	31
Without phonophobia (%)	59^b^	33
Little or no functional disability (%)	69^b^	41

AAC-130, acetaminophen 500 mg, aspirin 500 mg, caffeine 130 mg per 2-tablet dose; PID, pain intensity difference from baseline

^a^Rescue medication was permitted at 2 h postdose

^b^ *P* < 0.001 versus placebo

^c^ *P* < 0.01 versus placebo

In a 3-arm, double-blind, parallel group study of migraine, Goldstein and coworkers (2006) treated 1555 patients with a fixed combination of AAC (acetaminophen 500 mg, acetylsalicylic acid 500 mg, and caffeine 130 mg), IBU 400 mg or placebo. In comparison with placebo-treated participants, both active treatment arms demonstrated better relief of pain and associated symptoms. AAC was superior to IBU for a number of endpoints, including TOTPAR at 2 h, time to onset of meaningful PAR, pain intensity reduction, headache response, and pain free. The mean TOTPAR2 scores were 2.7 for AAC, 2.4 for IBU, and 2.0 for placebo (AAC vs IBU, *P* < 0.03). The median time to meaningful PAR for AAC was 20 min earlier than that of IBU (*P* < 0.036). The influence of caffeine once again could not be assessed as this was not a factorial study [114].

#### Efficacy in mixed populations (TTH and migraine)

Two studies evaluated the efficacy of analgesics and caffeine in study populations that included patients with TTH and migraine [33, 115]. In the first, Diener et al. studied AAC-100 (APAP 200 mg, ASA 250 mg, and caffeine 50 mg), which is available OTC in Germany, in patients with TTH and migraine using a 6-arm, randomized, double-blind, parallel-group factorial design. The fixed combination AAC-100 was compared with APAP 200 mg and ASA 250 mg, APAP alone, ASA alone, caffeine alone and with placebo in 1743 subjects who typically treated their headaches with OTC medications [33]. Pain intensity was recorded on a 100-mm visual analogue scale. Among enrolled subjects, 84% had migraine, 13% had episodic TTH, and 3% could not be classified. The primary endpoint was time to 50% pain relief (PAR).

Time to 50% PAR, AAC-100 was significantly better than the APAP 200 mg-ASA 250 mg combination (*P* = .02), ASA 250 mg (*P* = .04), APAP 200 mg (*P* = .002), caffeine 50 mg (*P* < .0001), and placebo (*P* < .0001). All active treatments, except caffeine 50 mg, were significantly superior to PLA (*P* < .0001) [33]. The median time to 50% PAR was 65 min with AAC-100, 73 min with APAP 200 mg-ASA 250 mg, 79 min with ASA 250 mg, 81 min with APAP 200 mg, 107 min with caffeine 50 mg, and 133 min with placebo. Additional efficacy results comparing AAC-100 with the analgesic combination without caffeine and its constituents are shown in Table 4 [33]. It was concluded that AAC-100 significantly outperformed all the other treatments for reductions in pain intensity, and that the clinically meaningful improvements compared with APAP or ASA monotherapy confirmed the existence of an adjuvant effect of caffeine in migraineurs. Diener et al. also pointed out that, because their patient population typically treated their headache attacks with OTCs, findings can be generalized to the larger population of migraineurs and patients with TTH [33].

**Table 4:** Results of a 6-arm factorials study of the acute treatment of migraine and TTH^a^ [33]

Measure	AAC-100	APAP + ASA	APAP	ASA	Caffeine	Placebo
PID @ 2 h (mm)	44.7	40.2	39.5	40.7	31.4	24.6
Active vs placebo	<0.0001	<0.0001	<0.0001	<0.0001	<0.02	–
Active vs AAC-100	–	0.002	0.02	0.003	<0.0001	<0.0001
Weighted SPID (%)	66.6	62.0	57.5	62.2	46.7	40.2
P vs placebo	<0.0001	<0.0001	<0.0001	<0.0001	0.0993	–
P vs AAC-100	–	0.02	0.07	0.0002	<0.0001	<0.0001
Functional disability @ 2 h (%)	53.9	49.4	48.6	48.4	39.4	30.5
P vs placebo	<0.0001	<0.0001	<0.0001	<0.0001	0.1491	–
P vs AAC-100	–	0.08	0.08	0.04	<0.0001	<0.0001
Global assessment of efficacy (%)	25.2	21.4	15.4	18.7	20.8	10.9
P vs placebo	<0.0001	<0.0001	<0.0001	<0.0001	0.0132	–
P vs AAC-100	–	0.01	<0.0001	0.009	<0.0001	<0.0001

AAC-100, acetaminophen 400 mg, aspirin 500 mg, caffeine 100 mg per 2-tablet dose; APAP + ASA, acetaminophen 400+ aspirin 500 mg per 2-tablet dose; APAP, acetaminophen 400 mg per 2-tablet dose; ASA, aspirin 500 mg per 2-tablet dose; caffeine, caffeine 100 mg per 2-tablet dose; PID, pain intensity difference from baseline; SPID, sum of pain intensity differences

^a^The study population included patients with migraine (84%) and TTH (13%) who typically treated attacks with non-prescription analgesics

#### Efficacy in children and adolescents with migraine or TTH

A double-blind, crossover pilot study comparing the efficacy of an IBU-caffeine combination with an IBU-placebo combination in 12 children (7 girls, 5 boys) aged 7 to 15 years (mean = 11.9 years) who had TTH (25%) or migraine (75%) was conducted [116]. Study treatments, adjusted to subjects’ weight, included 100 mg IBU + 50 mg caffeine; 200 mg IBU + 50 mg caffeine; 300 mg IBU + 100 mg caffeine; or 400 mg IBU + 100 mg of caffeine. Baseline pain intensity, which was assessed on a 4-point scale (4 = severe, 3 = moderate, 2 = mild, 1 = no pain), was 3.8 in the IBU-caffeine treatment group and 4.0 in the IBU-placebo group [116].

A total of 58% of children obtained faster relief with the IBU-caffeine combination, while 33% appeared to achieve earlier relief with IBU and placebo, and 8% exhibited no difference in the response [116]. Time to meaningful relief (pain intensity ≤2) was about twice as fast (60 min) for attacks treated with IBU-caffeine as it was for those treated with IBU-placebo (≈120 min). At 30 min postdose, PID in the IBU-caffeine group was 1.0 compared with 0.2 in the IBU-placebo group; at 60 min postdose, PID scores were 1.5 for IBU-caffeine and 0.9 for IBU-placebo. Although the IBU-caffeine combination failed to separate significantly on any efficacy endpoint in this small pilot study, it was numerically superior to the IBU-placebo combination, and almost 60% of patients realized clinically important benefits [116].

### Tolerability

In these studies, ≥96% of patients reported adverse events (AEs) [33, 34, 73, 74]. AEs were usually mild or moderate, and no subject in any study withdrew as a result of an AE that was considered to be related to active treatment. Across all studies and patient types, the most commonly reported treatment-emergent AEs were nervousness (6.5% [4.0–11.0%]), nausea (4.3% [1.0–7.0%]), abdominal pain/discomfort (4.1% [1.1–9.0%]), and dizziness (3.2% [0.8–6.0%]) [33, 34, 73, 74].

### Estimate of benefit

Previous meta-analyses have estimated that combining analgesics and/or NSAIDs with caffeine improves antinociception. In patients with a range of pain conditions, Laska et al. suggested an adjuvant effect of 1.42 for AAC-130 compared with APAP or ASA monotherapy [32]. Migliardi et al. estimated an adjuvant benefit of 1.63–1.97 for AAC-130 versus APAP in patients with TTH [74]. Zhang found an effect of 1.36 (95% CIs 1.17–1.58) for APAP-caffeine combinations versus APAP alone in patients with headache [75]. A Cochrane review of caffeine adjuvancy in various pain states reported that the number needed to treat (NNT) for an additional patient to have relief of headache pain was 14 while the NNTs for postoperative dental pain, postoperative pain, and dysmenorrhea pain were 13, 16, and 25, respectively. Considered by dose, 65 mg or less had no adjuvant effect in postoperative pain, while the NNT for doses of 70–150 mg was 14, and for doses above 150 mg, the NNT was 10.^164^ Overall, there appeared to be a small but clinically significant benefit to adding caffeine to analgesic therapy for various types of acute pain, but more research is needed to optimize dosing recommendations [117].

## Discussion

This review provides evidence for the role of caffeine as an analgesic adjuvant in the acute treatment of primary headache with OTC drugs. A dose of 130 mg enhances the efficacy of OTC analgesics in TTH and a dose of ≥100 mg enhances the benefits of OTC analgesics in migraine. The incidence of treatment-emergent AEs in these studies was low, and the type and severity of AEs was similar across headache diagnoses. It seems likely that abdominal pain was probably due to the presence of NSAIDs (ie, ASA or IBU), while nervousness, nausea, and dizziness were most likely related to consumption of caffeine. These AEs, none of which were severe or unexpected, suggest that most patients who use caffeine-containing OTC combinations for occasional, acute treatment of TTH or migraine will experience good tolerability with appropriate use.

The relationship between caffeine and headache is complex, paradoxical, and often misunderstood. Used appropriately, caffeine significantly enhances the effectiveness of analgesics and NSAIDs in the treatment of patients with migraine and TTH. Used to excess, caffeine-containing analgesics can place patients at risk of medication overuse headache (MOH) and the progressive development of chronic TTH or chronic migraine [1]. At the same time, results from an uncontrolled, clinic-based study suggest that discontinuing caffeine consumption can improve the efficacy of acute migraine treatment [83].

Caffeine also has intrinsic antinociceptive properties, which enable it to be used as a monotherapy for relief of hypnic headache. However, large acute doses can precipitate headache, as can abrupt cessation after regular dietary consumption. On the other hand, if addition of caffeine improves efficacy, it may reduce the number of doses of acute medication need to successfully treat an attack of migraine. In addition, patients are often willing to use OTC products earlier in an attack of headache which likely improves outcome and may reduce need for further treatment. The risk of MOH is one of frequency of use.

Appropriate dosing of caffeine in patients with migraine or TTH remains uncertain. Laska et al. suggest that a caffeine dose of 130 mg may be optimal for achieving the analgesic adjuvant effect among patients with headache [32], as lower doses (<60 mg) generally do not show a reliable adjuvant effect. Further, if low doses of caffeine inhibit antinociception, dietary caffeine might interfere with analgesic efficacy [31].

Awareness of the role of caffeine in the management of patients with headache should facilitate optimal use and help to avoid or address MOH. In clinical practice, certain groups of patients may benefit, including those with a partial response to simple analgesics and migraineurs with prominent gastroparesis-related nausea.

## Conclusions

Caffeine is widely consumed around the world in both food and beverages, and it has a variety of important medical applications. In patients with headache disorders, caffeine monotherapy may be useful in some forms of primary or secondary headache. Its principal role is as an adjuvant in fixed combinations with analgesic medications for acute treatment of TTH and migraine. Evidence from clinical trials in these patient populations indicates that combining caffeine with OTC analgesic medications, such as APAP, ASA, and IBU, significantly improves efficacy over the analgesic alone. As might be expected with OTC preparations, tolerability is good for the vast majority of patients, and AEs are predictable and almost universally mild and transient. Additional studies are needed to assess the relationship between caffeine dosing and clinical benefit in patients with TTH and migraine.

## Abbreviations

AAC-100: APAP 400 mg, ASA 500 mg, and caffeine 100 mg
AAC-130: APAP 500 mg, ASA 500 mg, and caffeine 130 mg
AE: Adverse event
APAP: Acetaminophen
ASA: Acetylsalicylic acid
GRAS: Generally recognized as safe
IBC-100: IBU 400 mg and caffeine 200 mg
IBU: Ibuprofen
MOH: Medication overuse headache
NNT: Number needed to treat
NSAID: Nonsteroidal anti-inflammatory drugs
OTC: Over the counter
PAR: Pain relief
PDPH: Post-dural puncture headache
PID: Pain intensity difference
SPID: Summed pain intensity difference
TOTPAR: Total pain relief
TTH: Tension-type headache
